# Phosphorylation of PP2Ac by PKC is a key regulatory step in the PP2A-switch-dependent AKT dephosphorylation that leads to apoptosis

**DOI:** 10.1186/s12964-024-01536-7

**Published:** 2024-02-28

**Authors:** Guy Nadel, Zhong Yao, Avital Hacohen-Lev-Ran, Ehud Wainstein, Galia Maik-Rachline, Tamar Ziv, Zvi Naor, Arie Admon, Rony Seger

**Affiliations:** 1 Department of Immunology and Regenerative Biology, the Weizmann Institute of Science, Rehovot, Israel; 2 Smoler Proteomic Center, Technion–Israel Institute of Technology, Haifa, Israel; 3 Department of Biochemistry and Molecular Biology, Tel Aviv University, Tel Aviv, Israel; 4 Faculty of Biology, Technion-Israel Institute of Technology, Haifa, Israel

**Keywords:** PP2A phosphorylation, PP2A switch, PKC, JNK, Apoptosis

## Abstract

**Background:**

Although GqPCR activation often leads to cell survival by activating the PI3K/AKT pathway, it was previously shown that in several cell types AKT activity is reduced and leads to JNK activation and apoptosis. The mechanism of AKT inactivation in these cells involves an IGBP1-coupled PP2Ac switch that induces the dephosphorylation and inactivation of both PI3K and AKT. However, the machinery involved in the initiation of PP2A switch is not known.

**Methods:**

We used phospho-mass spectrometry to identify the phosphorylation site of PP2Ac, and raised specific antibodies to follow the regulation of this phosphorylation. Other phosphorylations were monitored by commercial antibodies. In addition, we used coimmunoprecipitation and proximity ligation assays to follow protein–protein interactions. Apoptosis was detected by a TUNEL assay as well as PARP1 cleavage using SDS-PAGE and Western blotting.

**Results:**

We identified Ser24 as a phosphorylation site in PP2Ac. The phosphorylation is mediated mainly by classical PKCs (PKCα and PKCβ) but not by novel PKCs (PKCδ and PKCε). By replacing the phosphorylated residue with either unphosphorylatable or phosphomimetic residues (S24A and S24E), we found that this phosphorylation event is necessary and sufficient to mediate the PP2A switch, which ultimately induces AKT inactivation, and a robust JNK-dependent apoptosis.

**Conclusion:**

Our results show that the PP2A switch is induced by PKC-mediated phosphorylation of Ser24-PP2Ac and that this phosphorylation leads to apoptosis upon GqPCR induction of various cells. We propose that this mechanism may provide an unexpected way to treat some cancer types or problems in the endocrine machinery.

**Supplementary Information:**

The online version contains supplementary material available at 10.1186/s12964-024-01536-7.

## Background

Protein phosphorylation is an important post-translational modification that plays a role in the regulation of cellular processes, either without or after stimulation [1, 2]. This is a reversible mechanism that is mediated by protein kinases [3] and protein phosphatases [4] that collectively maintain the dynamic phospho-proteome in all cells and under all conditions. Among other processes, phosphorylation is known to regulate most parts of cellular signaling, in which protein kinases usually mediate the “on” signal, while protein phosphatases, which are usually negative regulators, silence it and maintain low basal phosphorylation in non-stimulated cells [5]. However, in some cases, the phosphatases play important roles in enhancing cell-signaling and their downstream effects [6]. These type of “on” activities by phosphatases are generally ill-studied and more information on their nature is still required.

One of the main phosphatases that regulates cellular signaling is protein phosphatase 2A (PP2A), which dephosphorylates many signaling components and transcription factors [7, 8]. This is a protein Ser/Thr phosphatase that plays a role in the regulation of many cellular processes. It usually operates as a heterotrimer consisting of a scaffold (A subunit, PP2Aa), regulatory subunit (B) and catalytic subunit (C subunit, PP2Ac) [9, 10]. In humans, there are two distinct although very similar A subunits, two very similar C subunit, and 17 different B subunits, which determine the phosphatase specificity. In some cases, PP2A can also act as heterodimers either of PP2Ac and PP2Aa subunits without B subunit [7, 11], or PP2Ac with B subunits [12]. In addition, PP2Ac forms a dimer with other non-canonical subunits that may regulate their specificity and stability (e.g. IGBP1; also known as α4 [13, 14], and PTPA [15]). These distinct complexes usually demonstrate different substrate specificity and stability, and therefore have variable effects on cell fates in divergent cell types and conditions. Notably, certain compositions of PP2A were shown to take part in several pathologies. Particularly, it was shown that some of its complexes can act as tumor suppressors in some cells, while the dysregulation of this complexes leads to oncogenesis as well as other diseases [16–19].

In previous studies we showed that PP2A plays a pivotal role in the induction of apoptosis upon stimulation of Gq protein coupled receptors (GqPCRs) that act via PKCs, both in cancer and non-transformed cells [20–22]. The resulted apoptosis is mediated by PP2A-dependent reduction in the activity of the survival signaling mediator, AKT [23–25]. Indeed, it was previously shown that PP2A dephosphorylates both activatory p-Ser473 and p-Thr308 of AKT, to induce its full inactivation [23]. The B subunits that direct PP2A to AKT in mammals are usually PR56β and PR56γ [26], as well as B55 [27, 28], which can determine the proper and timely inactivation of AKT. However, we noticed that these B subunits are not involved in the GqPCR or PKC induced AKT inactivation in our system [22].

In order to further study the mechanism of GqPCR-induced inactivation of AKT, we have previously screened 21 cell lines and found a stimulated reduction in AKT phosphorylation in 10 of them [21]. This effect was PKC-dependent, correlated with reduced AKT activity, JNK activation, and in some cases led to apoptosis. We showed that the apoptosis is mediated by two signaling branches, converging at the level of MLK3, upstream of JNK. One branch consists of c-Src activation of MLK3, and the second includes reduction in AKT activity that alleviates its inhibitory effect on MLK3. We further identified the non-canonical B subunit, IGBP1 (α4), as a main regulator of this process [22]. In resting cells, an IGBP1-PP2Ac dimer binds to PI3K and dephosphorylates the inhibitory pSer608-p85 subunit of PI3K to maintain its high basal activity. Upon GqPCR activation, the PP2Ac-IGBP1 dimer detaches from PI3K and thus allows the inhibitory autophosphorylation of PI3K. Then, a PP2Ac-IGBP1-PP2Aa trimer binds to AKT, and causes its dephosphorylation and inactivation, acting as a PP2A switch. Thus, we delineated a general mechanism that mediates a GqPCR-induced, death receptors-independent, apoptosis that is mediated by a stimulated shift of PP2Ac from PI3K to AKT.

In the current manuscript we studied the molecular mechanism that induces the PP2A switch. Using Mass spectrometry (MS), we found a novel phosphorylation site at Ser 24 residue of PP2A catalytic subunit induced by GqPCR activation or by TPA stimulation. We raised specific antibodies (Abs) against the phosphorylated residue and generated phosphomimetic and unphosphorylatable mutations of this site to study the role of this phosphorylation in the PP2A switch. We found that phosphorylation of PP2Ac on Ser 24 is essential to regulate detachment of the PP2Ac from PI3K. We further found that the phosphorylation is mediated directly by PKCα/PKCβ phosphorylation and is necessary and sufficient to induce apoptosis. Our results show that the GqPCR-activated PP2A switch is regulated by PKC-mediated phosphorylation of Ser24 in PP2Ac, that ultimately leads to JNK-mediated apoptosis.

## Methods

### The aim, design and setting of the study

The aim of this study was to elucidate the mechanism by which the AKT-inactivating PP2A switch is initiated and regulated. In order to do so, we used phospho-MS to identify Ser24 as a phosphorylation site on PP2Ac. We further found that this site is phosphorylated by classical PKCs upon GqPCR stimulation. Finally, we used phosphomimetic and unphosphorylatable mutants of PP2Ac to show the importance of the phosphorylation in inducing AKT inactivation, JNK activation and apoptosis.

### Reagents and antibodies

GnRH analog (GnRH-a), Tetradecanoylphorbol acetate (TPA), Polyethylenimine (PEI), 4′6-diamino-2-phenylindole (DAPI) and PLA kit were obtained from Sigma (Rehovot, Israel). GF109203x (GFx) was obtained from Calbiochem (Darmstadt, Germany). Protein A/G beads were obtained from Santa Cruz Biotechnology (Santa Cruz, CA, USA). Dharmafect was obtained from Thermo Scientific (Lafayette, CO, USA). 8-iso PGF2α was purchased from Cayman Chemical (Ann Arbor, MI, USA). Monoclonal anti-PP2Ac Ab was obtained from BD Transduction Laboratories (New Jersey, USA; catalog # 610,556, Lot 0149933). Anti-IGBP1 (catalog # ab70545), Tubulin (catalog # ab21057) and monoclonal anti-p85-PI3K (catalog # ab86714) Abs were obtained from Abcam (Cambridge, UK). Anti-HA (catalog # 11,867,423,001) and GFP (catalog # 11,814,460,001) Abs were obtained from Roche Diagnostics (Mannheim, Germany). PKCα (catalog # sc80) and PP2Aa (catalog # sc13600, Lot L1319) were obtained from Santa Cruz Biotechnology (CA, USA). Abs to phosphorylated JNK (pJNK) (catalog # J4750, Lot 123M4892), general JNK1/2 (gJNK) (PT48, catalog # FXO11038 Lot 21,070,625), and pFOXO1 (catalog # PA5, Lot 38,132) were obtained from Sigma (Rehovot, Israel). Anti-phospho AKT (pS473AKT) (D9E, catalog # 4060), Pan AKT (catalog # 4691, Lot 28) and PARP1 (catalog # 9542, Lot15) were obtained from Cell Signaling Technology (Boston, MA, USA). The anti-phosphorylated PP2A (S24) and anti-phosphorylated PI3K (S608) Abs were prepared by the Ab unit of the Weizmann Institute of Science (Rehovot Israel) as described [22]. Secondary Ab were from Jackson Immunoresearch (West Grove, PA, USA). IgG Ab (catalog # sc69786, Lot K0521) was obtained from Santa Cruz Biotechnology (CA, USA).

### Buffers

Buffer A: 50 mM β-glycerophosphate (pH 7.3), 1.5 mM EGTA, 1 mM EDTA, 1 mM dithiothreitol, and 0.1 mM sodium vanadate. Buffer H: 50 mM β-glycerophosphate, pH 7.3, 1.5 mM EGTA, 1 mM EDTA, 1 mM DTT, 0.1 mM sodium vanadate, 1 mM benzamidine, 10 µg/ml aprotinin, 10 µg/ml leupeptin, and 2 µg/ml pepstatin A. Coimmunoprecipitation (CoIP) buffer: 20 mM HEPES pH 7.4, 2 mM MgCl_2_, 2 mM EGTA, 150 mM NaCl and 0.1% Triton.

### Cell culture and transfection

αT3 cells were obtained and cultured as previously described in [29]. Briefly, the cells were cultured in Dulbecco’s modified Eagle’s medium (DMEM) supplemented with 2 mM L-glutamine, 1% pen/strep and 10% fetal calf serum (FCS). SVOG4 cells from N. Auersperg (University of British Columbia, Vancouver, Canada) were cultured in the same medium combination with the addition of hydrocortisone (0.5 mg/ml), and Gentamycin Hydrochloride. PC3 cells from ATCC were cultured in RPMI supplemented with 2 mM L-glutamine, 1% pen/strep and 10% FCS. αT3-1 or PC3 cells were transfected by adding polyethylenimine (Sigma; Rehovot Israel [30]) and the examined plasmid and the cells were grown in a starvation medium (0.1% FCS 16 h). The simultaneous transfection and starvation was used to save time to avoid the apoptotic effects that start ~ 30 h after transfection when the S24E plasmid is expressed. SiRNAs were transfected using Dharmafect (Dharmacon) according to the manufacturer’s instructions.

### Plasmids

PP2Ac plasmid was cloned from mRNA from HeLa cells into HA-pCDNA3 between the HindIII and BamHI sites. PP2Aa cDNA in pMIG was obtained from Addgene, and transferred into pEGFPC1 between EcoRI and KpnI with FLAG preserved. The mutants were prepared using Quickchange method Agilent (Santa Clara CA, USA).

### Cell extraction and Western blotting

Cells were grown to subconfluency and then serum-starved (0.1% FCS for 16 h, as described in [31]). After stimulation or other treatments, cells were rinsed twice with ice-cold phosphate buffered saline (PBS), which was replaced with Buffer H. The cells were then scraped into Buffer H (0.5 ml/plate), sonicated (50 W, 2 × 7 s), and centrifuged (15,000 × g, 15 min). Aliquots of cellular extracts were subjected to SDS-PAGE and transferred onto nitrocellulose membranes (Tamar, Jerusalem, Israel) by electroblotting. Membranes were incubated with the corresponding primary Ab (60 min, 23 °C), followed by washes and incubation with horseradish peroxidase conjugated secondary Ab. Blots were developed using the ChemiDoc (BioRad, Hercules, CA USA). Each experiment was performed at least three times to obtain significant data. Quantification of the band intensities was performed using BioRad analysis tool (Madison WI, USA).

### Coimmunoprecipitation

Cells were grown to sub-confluence, serum-starved as above, and treated as indicated. Cell extracts were produced as previously described [32] and incubated for 2 h at 4 °C with Protein A/G-agarose beads (Santa Cruz Biotechnology, CA, USA) pre-linked with specific Abs (1 h, 23 °C). The bound A/G beads were washed three times with ice-cold washing buffer containing 10 mM Tris, pH 7.4, 1 mM EDTA, 1 mM EGTA, pH 8.0, 150 mM NaCl, and 0.5% Triton X-100. Beads were then resuspended with 1.5X sample buffer and boiled; the resolved proteins were analyzed by Western blotting with the indicated Abs.

### Mass spectrometry

Serum-starved PC3 cells were stimulated with TPA (250 nM, 10 min), and then lysed. PP2A was immunoprecipitated (IPed) with anti-PP2Ac Ab and then further purified using SDS-PAGE. The PP2A components were then in-gel trypsinized and extracted from the proper region of the gel and were then subjected to LC–MS-MS analysis as follows: The proteins in the gel were reduced (3 mM DTT), modified with 12 mM iodoacetamide and trypsinized with modified trypsin (Promega, Madison WI, USA) at a 1:10 enzyme-to-substrate ratio, all in 10 mm ammonium bicarbonate and 10% acetonitrile. The resulting tryptic peptides were resolved by reversed-phase chromatography on 0.075 mm ID and about 20 cm long fused silica capillaries (J&W) packed with Reprosil-Aqua-C18, 3 µm beads (Dr Maisch GmbH, Germany). MS was performed with Q-Exactive-plus mass spectrometer (Thermo Fisher Scientific) in a positive mode using repetitively full MS scan followed by high collision induced dissociation (HCD) of the 10 most dominant ions selected from the full MS scan. The peptides were eluted with linear 60 min gradient of 5% to 28% acetonitrile with 0.1% formic acid in water 15 min gradient of 28% to 95% acetonitrile with 0.1% formic acid in water and min at 95% acetonitrile with 0.1% formic acid in water at flow rates of 0.15 μl/min. The MS data were analyzed using Proteome Discoverer 1.4 software using Sequest (Thermo Fisher Scientific) algorithm searching against the human Uniprot database and specific sequences. Minimal peptide length was set to six amino acids and a maximum of two miscleavages was allowed. Peptide- and protein-level false discovery rates (FDRs) were filtered to 1% using the target-decoy strategy. Semi quantitation was done by calculating the peak area of each peptide, based on its extracted ion currents (XICs).

### Proximity ligation assay

Protein–protein interactions were detected with Duolink PLA Kit (Olink Bioscience), according to the manufacturer’s protocol as described [33]. Briefly, cells were plated on glass coverslips, in 12 well plates fixed and permeabilized as described for immunofluorescence staining. The samples were then incubated with primary Abs against two proteins suspected to interact (60 min, 23 °C), and then incubated with specific probes according to manufacturer’s protocol, followed by DAPI staining to allow visualization of nuclei. The signal was visualized as distinct fluorescent spots by fluorescence microscopy (Olympus BX51, or spinning disc confocal Zeiss microscope both at × 40 magnification). Background correction, contrast adjustment and the quantification of the fluorescence signal were performed using Photoshop (Adobe) and ImageJ.

### TUNEL

To analyze apoptosis, sub-confluent PC3 and αT3-1 cells were plated on glass coverslips in 12 well plates under the standard culture conditions as described above. Twenty-four h after the initial seeding, cells were serum-starved and then treated. At different times after treatment the cells were fixed with paraformaldehyde solution (4% in PBS (pH 7.4) for 1 h at 15–25 °C), washed with PBS and then incubated with 0.1% Triton X- 100 in 0.1% sodium citrate (2 min, 4 °C), washed again with PBS, and incubated with terminal deoxynucleotidyltransferase-mediated nick end labeling (TUNEL) reaction mixture containing fluorescein-dUTP and terminal deoxynucleotidyltransferase (Roche Molecular Biochemicals Mannheim Germany) for 30 min at 37 °C. Preparations were analyzed by fluorescence microscopy.

### Statistical analysis

“Experiments with treatments and inhibitors: Values were compared using ANOVA, accounting for treatment, inhibitor, treatment-inhibitor interaction and repeat (batch). Time experiments: Values were compared using ANOVA, accounting for time and repeat. Pairwise comparisons were done using Tukey’s post-hoc test. All statistics were done using the program R, v. 4.3.1.”

## Results

### The PP2A switch is regulated by PKC

**Fig. 1:**
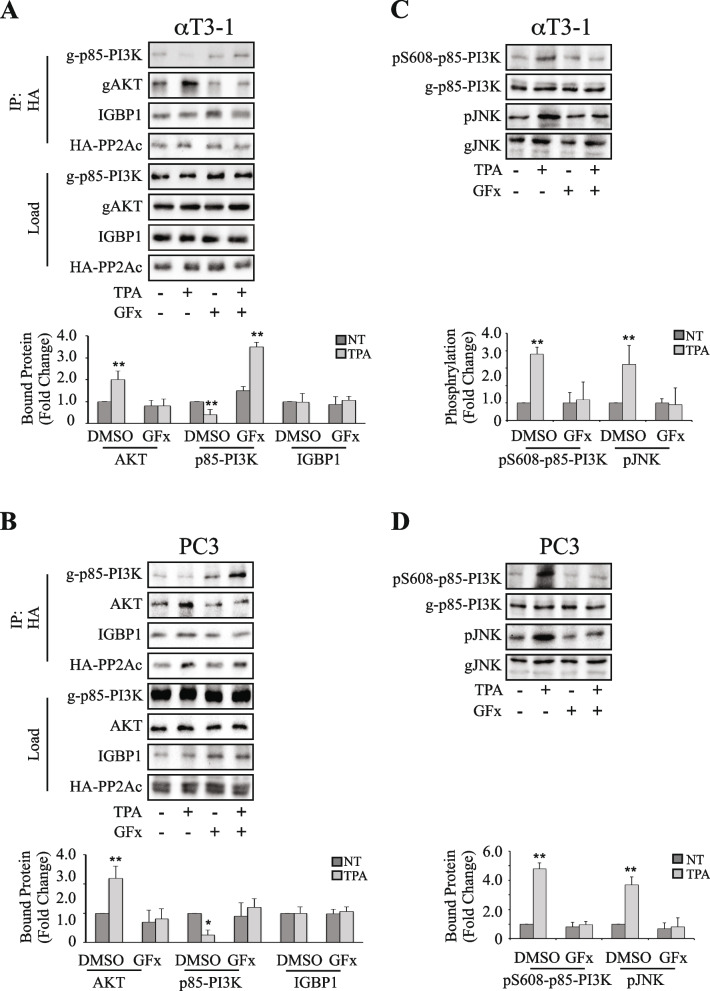
The PP2A switch-related interactions and phosphorylations are modulated by PKC inhibition. **A**, **B** Effect of PKC inhibition on PP2A switch-induced interactions. αT3-1 (A) or PC3 (B) cells were transfected with HA-PP2Ac (in PCDNA3) in serum-starvation medium (0.1% FCS, 16 h). The cells were then pretreated with PKC inhibitor GFx (3 µM, 20 min) or DMSO (control) and then were either stimulated with TPA (250 nM, 30 min) or left untreated. The cells were then harvested, and PP2Ac was IPed with anti-HA Ab. The Co-IPed PI3K, AKT and IGBP1 as well as the IPed HA-PP2Ac were detected by Western blotting with the relevant Abs. The bar-graphs in the lower panels represent means ± standard deviation (SD) of three experiments. Significance of change from DMSO, non-stimulated (-) control is calculated. * *p* < 0.01, ** *p* < 0.05. **C**, **D **Effect of PKC inhibitors on PP2A switch-induced phosphorylations. αT3-1 (C) or PC3 (D) cells were serum-starved (0.1% FCS, 16 h). The cells were then pretreated with PKC inhibitor GFx (3 µM, 20 min) or DMSO (control) and then either stimulated with TPA (250 nM, 30 min) or left untreated. Cells were harvested and the extracts were analyzed by Western blotting with the indicated Abs. The bar-graphs in the lower panels represent means ± SD of 3 experiments. Significance of change from DMSO, non-stimulated control (-) is calculated. * *p* < 0.01, ** *p* < 0.05

In the previous study [21], we found that stimulation of GqPCR results in inactivation of AKT downstream of PKC in certain cell lines. We have further shown that this is mediated by a PP2A switch which is mediated by changes in interactions of IGBP1, PP2Ac and PP2Aa with PI3K and AKT, resulting in the inactivation of the latter. Here we undertook to study the mechanism of regulation of the PP2A switch, and for this purpose, we used the αT3-1 and PC3 cell lines, that were previously identified as the best responders to the PP2A switch. First, it was important to confirm that the PP2A switch is indeed regulated by the activated PKC. For this purpose, we used the PKC inhibitor GF109203x (GFx) on several key players of the PP2A switch, and found that this inhibitor prevents the switch-related detachment of PP2Ac from PI3K, and the increased interaction of PP2Ac with AKT (Fig. 1A, B). As expected, the inhibitor did not affect the interaction of PP2Ac with IGBP1, supporting our previous hypothesis that this binding is persistent both before and after stimulation. We then examined the upstream inhibitory autophosphorylation of S608-p85-PI3K, which is antagonized by the bound PP2Ac. As expected by the inhibition of PP2Ac release from PI3K by GFx, the stimulated phosphate accumulation at this site was indeed prevented, inhibiting the downstream switch effect and finally preventing JNK phosphorylation (Fig. 1C, D). Taken together, these results show that PKC is indeed an upstream component, regulating the PP2A switch upon stimulation.

### PP2Ac is phosphorylated on Ser24-PP2Ac upon TPA stimulation

**Fig. 2:**
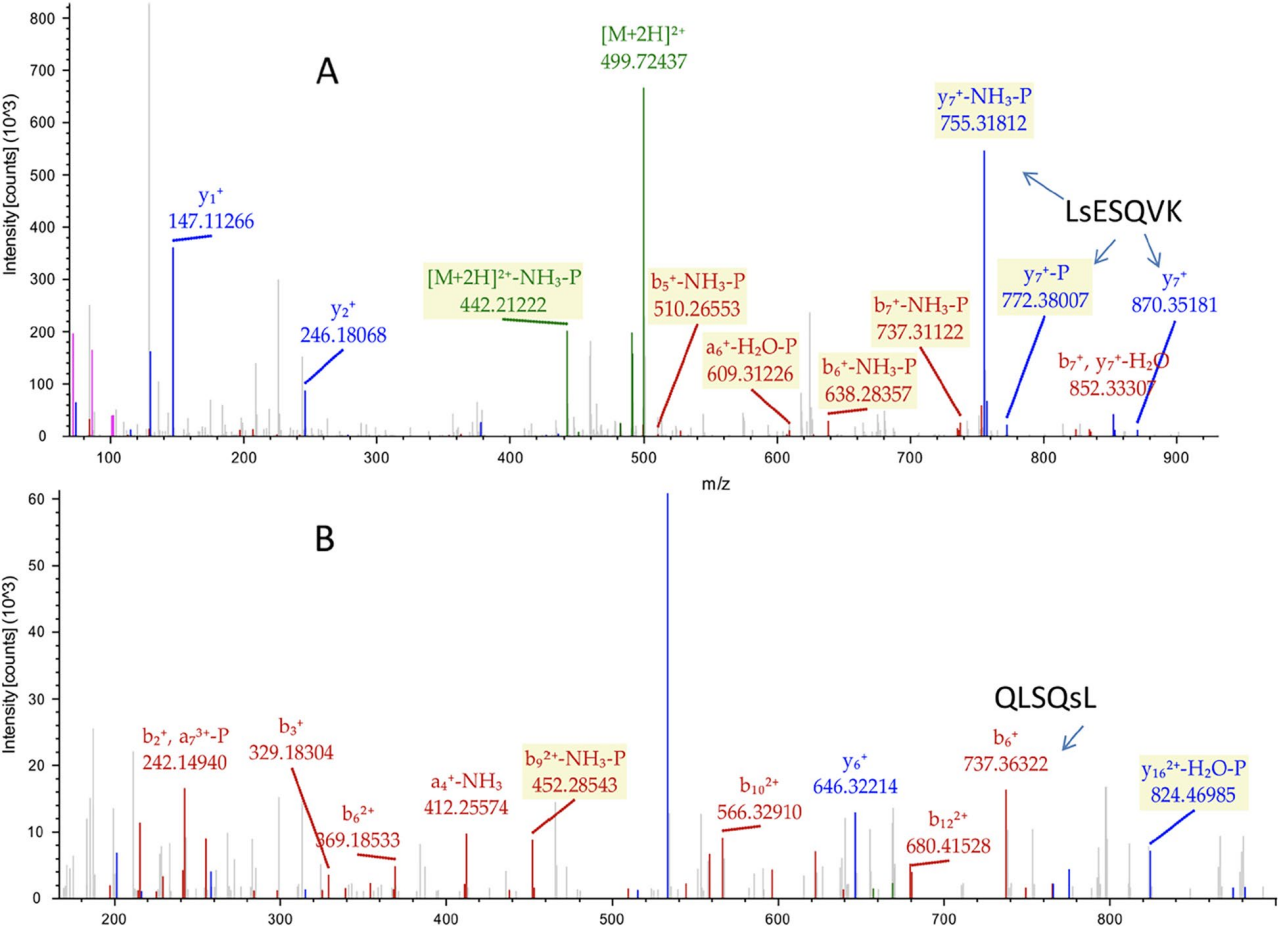
Identification of phosphorylation sites on PP2Aa and PP2Ac by MS. PC3 cells were serum-starved (0.1% FCS, 16 h). The cells were stimulated with TPA (250 nM, 30 min), and then endogenous PP2Ac was IPed using monoclonal anti-PP2Ac Ab (BD Transduction Laboratories), and subjected to LC–MS analysis. **A** HCD spectrum of the doubly charged ion (M/Z-499.72) interpreted as QLS(p)ESQVK phosphorylated S24 of PPP2Ac. **B** HCD spectrum of the triply charged ion (M/Z-669.013) interpreted as QLSQS(p)LLPAIVELAEDAK phosphorylated S403 of PPP2R1A. b ions are marked in red, y ion in blue, and the precursor in green

**Fig. 3:**
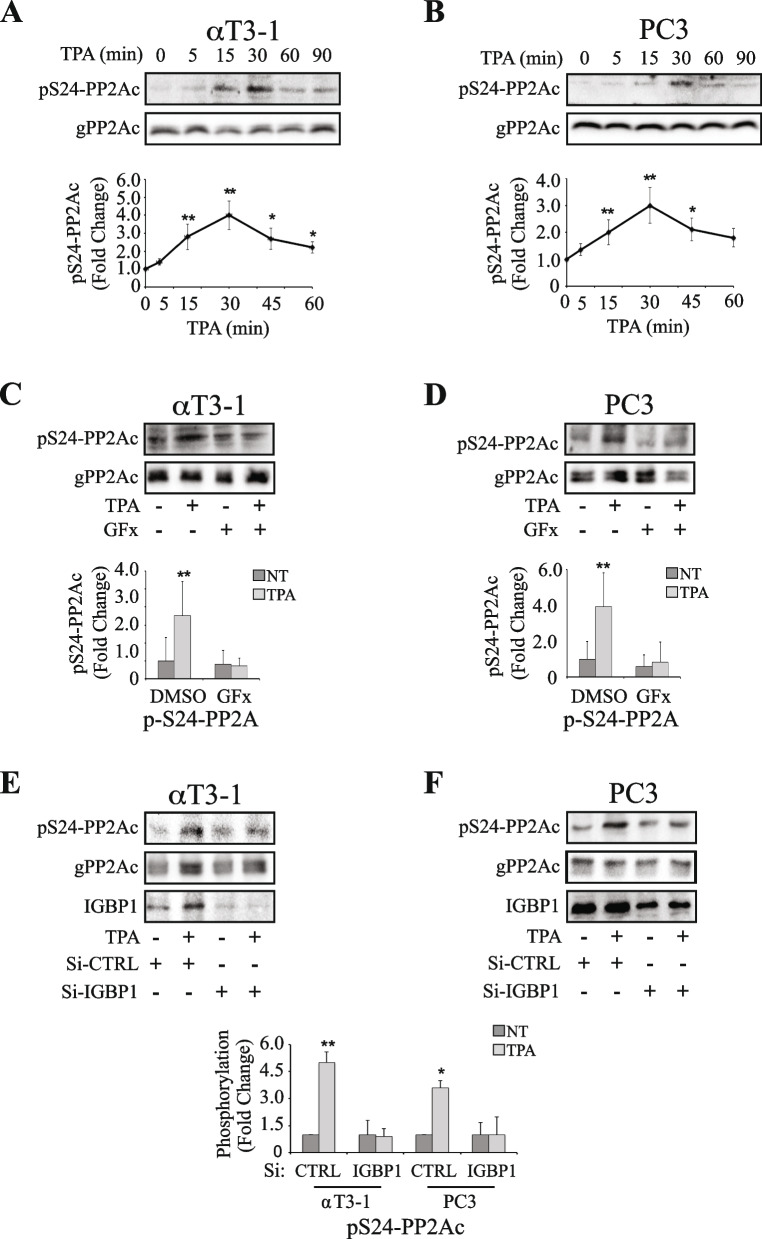
Ser24-PP2Ac is phosphorylated upon stimulation in a PKC and IGBP1-depndent manner. **A**, **B** Time course of S24-PP2Ac phosphorylation upon TPA stimulation. αT3-1 (A) or PC3 (B) cells were serum starved (0.1% FCS, 16 h), followed by stimulation with TPA (250 nM) for the indicated times. Then, the cells were harvested and subjected to Western blot analysis using an anti-phospho Ser24-PP2Ac raised for this study (see Methods). The lower graphs represent means ± SD of three experiments. Significance of change from non-stimulated (time 0) control is calculated. * *p* < 0.01, ** *p* < 0.05. **C**, **D** PKC is involved in Ser24-PP2Ac phosphorylation upon stimulation. Serum starved (0.1% FCS, 16 h) αT3-1 (C) or PC3 (D) cells were pretreated with PKC inhibitor GFx (3 µM, 20 min) or DMSO control and then either stimulated with TPA (250 nM, 30 min) or left untreated. Cells were harvested, followed by analysis of the cell extracts by Western Blotting with the indicated Abs. The bar-graphs in the lower panels represent means ± SD of 3 experiments. Significance of change from DMSO, non-stimulated (-) control is calculated. * *p* < 0.01, ** *p* < 0.05. **E**, **F **IGBP1 is involved in the TPA-induced Ser24-PP2Ac phosphorylation. αT3-1 (E) or PC3 (F) cells, were treated with SiRNA of IGBP1 or SiRNA control, serum starved (0.1% FCS, 16 h) and either stimulated with TPA (250 nM, 30 min) or left untreated. The cells were harvested, and extracts were analyzed with the indicated Abs. The lower bar-graphs are means ± SD of three experiments. Significance of change between non-stimulated SiRNA control (-) and the other stimulated and non-stimulated cells is calculated. * *p* < 0.01, ** *p* < 0.05

The involvement of PKC in the induction of PP2A switch raised the possibility that this process is regulated by phosphorylation of PP2Ac. In order to address this point, we resorted to phospho-protein MS analysis using IPed PP2Ac from TPA-stimulated PC3 cells. Interestingly, several potential phosphorylation sites were identified, one in the A subunit (α isoform, either Ser403 or, in a lower probability, Ser401 (, and one in PP2Ac (α isoform, Ser24; Fig. 2). We then mutated the three phosphorylation sites to either the non-phosphorylatable Ala or to the phosphomimetic Glu residues. Only the Ser24-PP2Ac mutants presented changes in AKT dephosphorylation when expressed in PC3 cells, while the other mutants had no effect (data not shown). Next, we undertook to characterize this phosphorylation and study its role in the PP2A switch. For this purpose, we first raised anti-phospho-Ser24-PP2Ac Ab (Fig. S1), which recognized a 35 kDa band corresponding to that of PP2Ac upon TPA stimulation, but not when the cells were treated with GFx (Fig. S1A). Moreover, no band was detected when the blotting was done in the presence of the antigenic peptide, but not in the presence of non-phosphorylated peptide, supporting the specificity of the Ab. The quality of the Ab was confirmed also by its lack of recognition of the exogenously expressed PP2Ac from TPA treated cells (Fig. S1B). Interestingly, the Ab did recognize to some extent the phosphomimetic S24E-PP2Ac mutant, indicating that the recognition relies on the acidity of the site. Using this specific Ab, we found that in both αT3-1 and PC3 cells, Ser24-PP2Ac is phosphorylated already 5 min after stimulation (Fig. 3A, B), peaks at 30 min and remains high up to 90 min after stimulation. We also found that the phosphorylation was significantly inhibited by the PKC inhibitor GFx (Fig. 3C, D), indicating again that this phosphorylation is regulated by PKC. Having demonstrated the role of IGBP1 in the switch [22], we examined the role of this regulator in the phosphorylation of Ser24, and found that knockdown of IGBP1 indeed inhibited this phosphorylation (Fig. 3E, F). This result indicates that the phosphorylation requires IGBP1 and therefore is related to the regulation of the PP2A switch. This could be due to the inability of IGBP1-lacking PP2Ac to attach to PI3K at the membrane [22], thus limiting the access of the Ser24-PP2Ac to the kinase. Taken together, these results confirm that Ser24-PP2Ac is being rapidly phosphorylated upon TPA treatment, and this phosphorylation affects the PP2A switch, so it may affect PP2A functions shortly after stimulation.

### Ser24-PP2Ac is phosphorylated by PKC

**Fig. 4:**
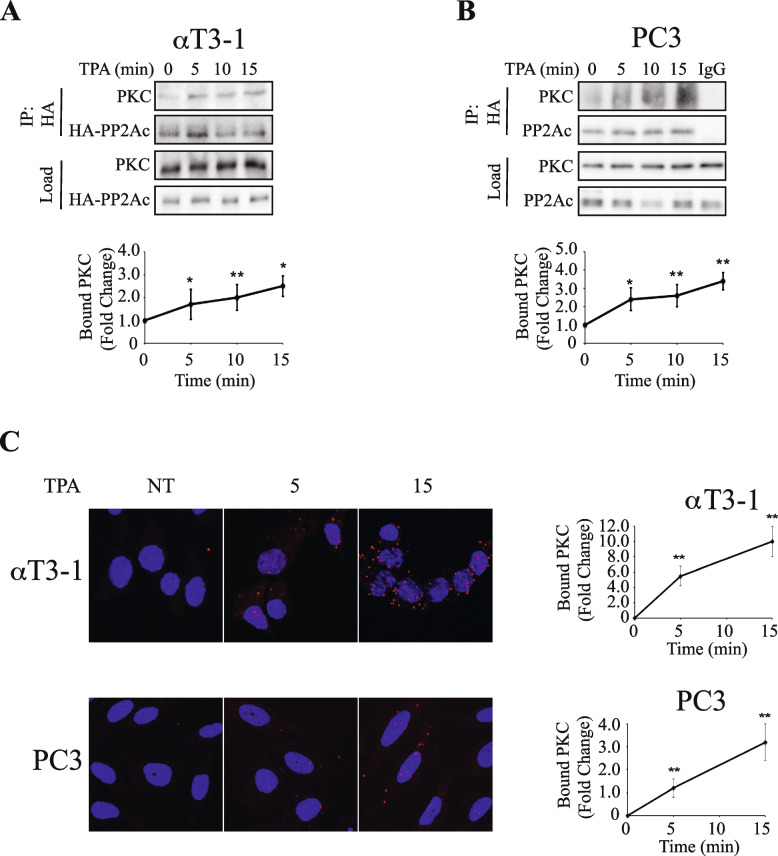
PKC directly interacts with PP2Ac. Determining PP2Ac-PKC interaction using CoIP. **A**,** B** αT3-1 (A) or PC3 (B) cells were transfected with HA-PP2Ac (in PCDNA3) in serum starvation medium (0.1% FCS, 16 h). The cells were then stimulated with TPA (250 nM) for the indicated times, harvested, and PP2Ac was IPed using anti-HA Ab. The CoIPed PKC, as well as the IPed HA-PP2Ac were detected by Western blotting with the relevant Abs. IgG was used as a CoIP negative control. The graphs in the lower panels represent means ± SD of 3 experiments. Significance of change from non-stimulated control (time 0) is calculated. * *p* < 0.01, ** *p* < 0.05. **C**. Proximity ligation assay (PLA) verifies PP2Aa interaction with PKC. αT3-1 (upper panel) and PC3 (lower panel) cells were cultured on cover slips, and were transfected with HA-PP2Ac (in PCDNA3) in serum starvation medium (0.1% FCS, 16 h). The cells were then either stimulated with TPA (250 nM, 5 or 15 min) or left untreated (both panels of αT3-1 cells and TPA 0 in PC3 cells) followed by fixation. Interactions of HA-PP2Ac and PKC were detected using PLA with their cognate Abs as described under Methods. The bar-graphs in the lower panels represent means ± SD of 3 experiments. Significance of change from non-stimulated control (NT) is calculated. * *p* < 0.01, ** *p* < 0.05

The results above clearly indicate that S24-PP2Ac is phosphorylated upon PKC activation. However, it was not clear whether this phosphorylation is mediated directly by one of the PKCs or by another downstream kinase. This question is particularly important, since the sequence surrounding the phosphorylation site (ECKQL**S**ESQVKS) does not exactly fit the consensus phosphorylation site of PKCs or any other known kinases, although the two lysins in the sequence may serve to some extent a recognition site for basic residues-directed kinases such as PKC. To study whether the phosphorylating kinase is indeed PKC, we first examined whether PKC can phosphorylate PP2Ac. For this purpose, we IPed active and inactive PKCα from PC3 cells and subjected it to in vitro phosphorylation with IPed PP2Ac as a substrate. Using the anti phospho-Ser 24 Ab we found that indeed PKCα from stimulated, but not from unstimulated cells, phosphorylates PP2Ac, indicating that the phosphorylated kinase may be PKCα (Fig. S2A). To further confirm it, we examined whether PKCα can interact with PP2Ac under the conditions used. As seen in Fig. 4 and S2B,C, the interaction of PP2Ac with endogenous PKC was low, but significantly increased shortly after stimulation of αT3-1 and PC3 cells with TPA (Fig. 4), or with GqPCR activating ligands (GnRH-a and PGF2α, Fig. S2 B,C). The interaction presented was confirmed using proximity ligation assay (PLA; Fig. 4C). We also examined whether other PKC isoforms expressed in αT3-1 cells [34] can interact with PP2Ac and participate in the phosphorylation, and found that PKCβ1 slightly interacted with PP2Ac upon stimulation, but PKCδ and PKCε did not (Fig. S2D). Since the other PKCs are not significantly expressed in the cells examined, there was no point to test their involvement. Our results suggest a role of classical PKCs in the phosphorylation of Ser24-PP2Ac, although we cannot rule out some involvement of other PKC-downstream kinases.

### Ser24-PP2Ac phosphorylation regulates AKT as well as PI3K phosphorylation

Next, we undertook to study the role of Ser24-PP2Ac phosphorylation in the regulation of PP2A switch and its downstream effects. For this purpose, we prepared phosphorylation-site mutants of S24-PP2Ac, including unphosphorylatable Ser24Ala (S24A) and the phosphomimetic Ser24Glu (S24E) mutations. The wild type (WT) HA-PP2Ac (HA-PP2Ac) and the two mutants were exogenously expressed in αT3-1 and PC3 cells. The constructs were reproducibly expressed in ~ 40% of the cell and the ratio of exogenously expressed protein level was about 80%-100% of the endogenous PP2Ac in the expressing cells (detected as ~ 40% in Western blots). The expression of the exogenous proteins was usually equal among the constructs. Importantly, the catalytic activity of the expressed mutants was similar to that of WT-PP2Ac as determined by in-vitro dephosphorylation of both AKT and para-nitrophenylphosphate (pNPP), both with and without TPA stimulation (Fig. S3). This result suggests that the putative effect of S24 phosphorylation is catalytic activity independent-and may be mediated by changes in protein–protein interactions with PP2Ac regulators such as B subunits or substrates.

**Fig. 5:**
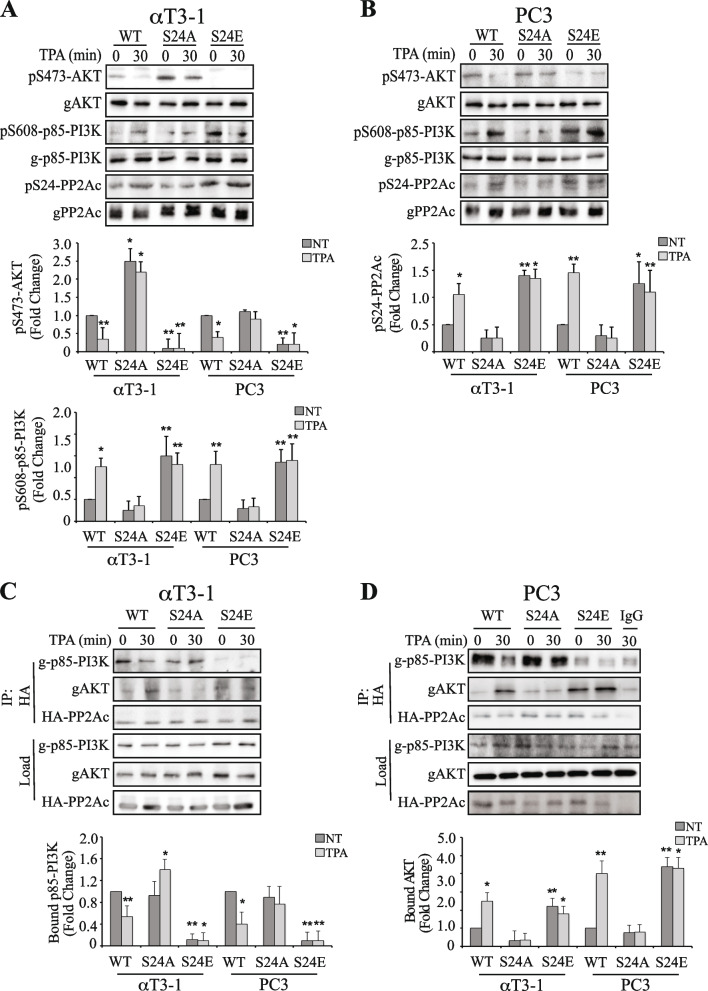
Ser24-PP2Ac phosphorylation regulates the PP2A switch through binding to PI3K and AKT but not IGBP1. **A**, **B** Ser24-PP2Ac phosphorylation regulates the PP2A switch related phosphorylation on AKT and PI3K inactivation. αT3-1 (A) or PC3 (B) cells were transfected with either HA-PP2Ac or PP2Ac mutants (S24A, S24E) in PCDNA3 in serum starvation medium (0.1% FCS 16 h). The cells were then either stimulated with TPA (250 nM, 30 min) or left untreated, harvested and analyzed using Western blot with the indicated Abs. The bar-graphs represent means ± SD of three experiments. Significance of change from non-stimulated control (time 0) is calculated. * *p* < 0.01, ** *p* < 0.05. **C**, **D** Ser24-PP2Ac phosphorylation regulates binding of PP2Ac to AKT and PI3K. αT3-1 (C) or PC3 (BD) cells were treated as described in panels A, B and PP2Ac was IPed using Ab against the HA tag. The CoIPed proteins were analyzed using the indicated Abs. IgG was used as a CoIP negative control. The bar-graphs represent means ± SD of three experiments. Significance of change from non-stimulated control (time 0) is calculated. * *p* < 0.01, ** *p* < 0.05

We then followed the effect of the transfected proteins on PP2Ac switch-related phosphorylations. As expected [21, 22], the phosphorylation of S473-AKT was significantly reduced upon TPA treatment in both cell lines (Fig. 5A, B). However, expression of S24A-PP2Ac abolished this reduction, while the expression of S24E-PP2Ac induced AKT dephosphorylation both before and after stimulation. These mutants affected not only pS473-AKT, but also the PP2A switch-related inhibitory autophosphorylation of Ser608 of PI3K. When HA-PP2Ac was expressed, there was a low S608 phosphorylation before stimulation (basal state), which was increased 30 min after stimulation. Expression of S24A-PP2Ac reduced the stimulated phosphorylation, while expression of S24E-PP2Ac elevated the basal phosphorylation, and this was not further elevated upon stimulation. Moreover, S24 phosphorylation nicely correlated with the phosphorylation of S608-p85-PI3K (Fig. 5A, B), suggesting that the effect on p85 might be downstream of Ser24 phosphorylation. Taken together, these results suggest that phosphorylation of Ser24-PP2Ac lies upstream of the Ser608-p85-PI3K phosphorylation, which may indicate that Ser24 phosphorylation is important for releasing PP2Ac from PI3K, an essential prerequisite step for the phosphate accumulation on Ser608 of P85-PI3K. Therefore, Ser24-PP2Ac phosphorylation is an essential step in the regulation of the PP2A switch.

**Fig. 6:**
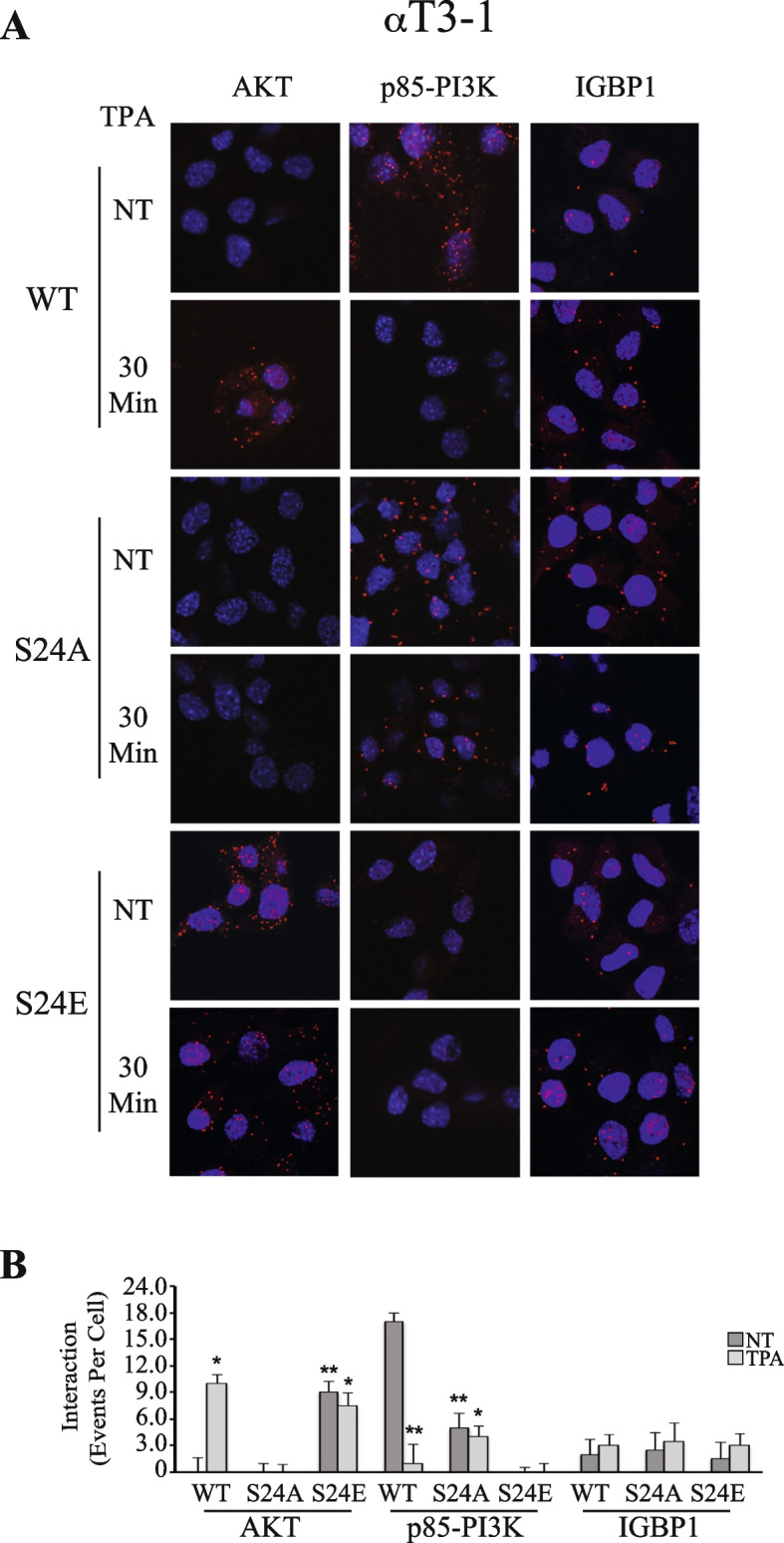
Using PLA to follow PP2Ac interaction with AKT, PI3K and IGBP1. **A** αT3-1 cells were cultured on cover slips. The cells were transfected with either HA-PP2Ac or PP2Ac mutants (S24A, S24E) in PCDNA3 in serum starvation medium (0.1% FCS 16 h). The cells were then either stimulated with TPA (250 nM, 30 min) or left untreated (NT) and fixed. Interactions were detected and quantified using the PLA kit, with anti HA for PP2Ac isoforms together with each of the other specific Abs (AKT, p85-PI3K and IGBP1) as described in the Method section. **B** The bar graphs represent means ± SD of a representative experiment that was reproduced 3 times. Significance of change from non-stimulated control (NT) is calculated. * *p* < 0.01, ** *p* < 0.05

Aside the significant effects of pSer24-PP2A mutations on the PP2A switch-related phosphorylations, we examined their effects on the switch-related interactions. As expected [22], the interaction of PP2Ac with PI3K was reduced upon stimulation, while the interaction with AKT was increased (Fig. 5C, D). Expressed S24A-PP2Ac did not significantly changed the interaction of the mutant with PI3K in basal state, and this was not significantly changed upon stimulation. Expression of S24E-PP2Ac resulted in a lower interaction of the mutant with PI3K and elevated interaction with AKT both before and after stimulation. The interaction results were confirmed using PLA in αT3-1 cells (Fig. 6) and PC3 cells (Fig. S4). Thus, expression of HA-PP2Ac in αT3-1 cells and applying anti PP2Ac plus anti-AKT Abs resulted in no PLA signal that did appear upon TPA stimulation. PI3K with HA-PP2Ac gave a strong signal in basal state, which disappeared upon stimulation. Moreover, similar to the CoIP results above, the PLA-detected interaction of PP2Ac with AKT was markedly reduced by the S24A mutation and elevated by the S24E mutations. The PLA-detected interaction of PP2Ac with PI3K was elevated by the S24A mutation and abolished by the S24E mutation. In these experiments (Figs. 6 and S4), we also examined the effects of the mutations on IGBP1-PP2Ac interaction. As expected [22], interaction between these proteins was readily detected without any change upon stimulation. Interestingly, the two mutants did not induce a significant change in the IGBP1-PP2Ac interaction, neither before or after stimulation. This result shows that Ser24-PP2Ac phosphorylation is important for PP2Ac detachment from PI3K but not for the IGBP1-induced interaction.

Another point that we addressed was the involvement of S24-PP2Ac phosphorylation in the interaction of PP2Ac-IGBP complex with PP2Aa when binding to AKT [22]. For this purpose, PC3 cells were transfected with GFP-IGBP1 and HA-PP2Ac constructs, grown in serum starvation medium and then were either stimulated with TPA or left untreated (0). IGBP1 was IPed from the extracts by anti GFP AB (precipitating mainly IGBP1-PP2Ac dimer), and the interaction of the endogenous PP2Aa was followed with Ab to this protein. The results (Fig. S5) indicate that there is no PP2Aa interaction with the IPed GFP-IGBP1, while TPA stimulation did cause such an interaction. Since IGBP1 cannot bind directly to PP2Aa, the most likely interpretation is that the observed interaction is not direct, and occurs via the PP2Ac. This conclusion is strengthened by the results with cells expressing the non-phosphorylated S24A-PP2Ac mutant that did not show any interaction with or without stimulation. Interestingly, the S24E-PP2Ac mutant did cause some interaction that was not changed after stimulation, indicating that the phosphomimetic residue allowed the (indirect, via PP2Ac) IGBP1-PP2Aa interaction. As discussed in our previous publication, there is a controversy in the literature regarding the feasibility of such an interaction, as these two proteins use a similar interaction site [22]. Our result here supports the notion that such a dual interaction does exist upon S24-PP2Ac phosphorylation. It is possible that this phosphorylation changes PP2Ac conformation to expose both sites in an angel that allows such a binding. Taken together, the CoIP as well as PLA results clearly indicate that the PP2A switch is significantly influenced by the phosphorylation of Ser24 residue of PP2Ac to allow detachment from PI3K and formation of a PP2Ac, IGBP1 and PP2Aa complex that binds to AKT.

### Phosphorylation of Ser24-PP2Ac is essential for JNK activation and apoptosis

**Fig. 7:**
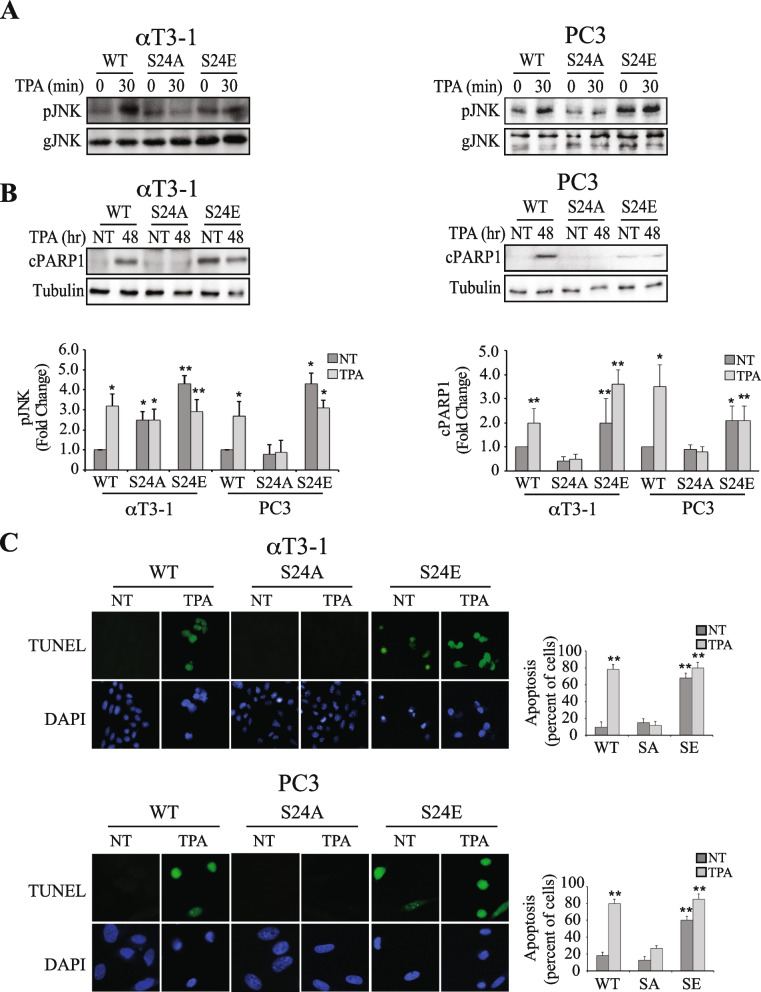
Ser24–PP2Ac phosphorylation induces JNK phosphorylation and apoptosis. **A** Ser24-PP2Ac phosphorylation is required for the PP2Ac switch-related JNK activatory phosphorylations. αT3-1 or PC3 cells were transfected with either HA-PP2Ac or PP2Ac mutants (S24A, S24E) in PCDNA3 in serum strvation medium (0.1% FCS 16 h). The cells were then either stimulated with TPA (250 nM, 30 min) or left untreated. Next, the cells were harvested, and cell extracts were analyzed by Western blotting with the indicated Abs. The bar-graphs in the lower panels represent means ± SD, of 3 experiments. Significance of change from non-stimulated HA-PP2Ac control (time 0) is calculated. * *p* < 0.01, ** *p* < 0.05. **B**, **C** Ser24-PP2Ac phosphorylation is required for the PP2Ac switch-induced apoptosis. Serum starved αT3-1 or PC3 expressing HA-PP2Ac or mutated PP2Ac as described in panel (**A**), were TPA (250 nM, 48 h) stimulated or left untreated. TPA-induced cell death was detected by cleaved PARP1 (**B**) or TUNEL (**C**). The results shown represent means ± SD. Significance of change from HA-PP2Ac, non-stimulated control (NT) was calculated. * *p* < 0.01, ** *p* < 0.05

We then studied whether the PKC-mediated Ser24 phosphorylation is essential and sufficient to promote the downstream effects of PP2A switch, primarily apoptosis. For this purpose, we used again the HA-PP2Ac as well as the S24A- and S24E-PP2Ac mutants in the examined αT3-1 and PC3 cells. We then examined the S24-PP2Ac dependent phosphorylation of Foxo1 that is a known AKT target upon stimulation [33, 35]. In agreement to AKT phosphorylation, the relatively high basal phosphorylation of Foxo1 was reduced upon stimulation in cells expressing HA-PP2Ac (Fig. S6). The S24A-PP2Ac mutant abolish this reduced phosphorylation, while the S24E-PP2Ac mutant increased FOXO1 phosphorylation in both non-stimulated and stimulated cells. Thus, this result show that the effect of pSer24-PP2Ac on AKT phosphorylation is transduced downstream to regulate the AKT-dependent pathway. We further examined JNK phosphorylation in both αT3-1 and PC3 cells exogenously expressing HA-PP2Ac and its mutants. In both cell lines, the S24A-PP2Ac mutant inhibited the phosphorylation of JNK, while the S24E-PP2Ac elevated it even in non-stimulated cells (Fig. 7A). Corresponding effects in the same cells were found for apoptosis detected 48 h post stimulation in both cells by detection of cleaved PARP1, a canonical marker of apoptosis (Fig. 7B), or using TUNEL assay (Fig. 7C,D). As expected from the JNK activation results, expression of S24A-PP2Ac abolished this induction of apoptosis, while S24E-PP2Ac induced a strong apoptosis signal even without stimulation. The results clearly indicate that S24E-PP2Ac expression by its own is sufficient to induce a significant apoptosis of the cell lines examined. Therefore, this phosphorylation is the first and most important regulatory step in regulating PP2A switch. This is done by inducing PKC-dependent signaling downstream the PP2A switch, depicting a necessary and sufficient role for PKC phosphorylation of PP2Ac in the induction of GqPCR-induced apoptosis.

## Discussion

The serine/threonine protein phosphatase PP2A heterotrimer complex regulates many cellular processes by dephosphorylation of a large number of regulatory proteins [7, 8]. In our previous studies we have shown that in 11 out of 21 cell lines examined, GqPCRs induces dephosphorylation and inactivation of AKT [21]. A common denominator of the responsive cells was high basal AKT phosphorylation/activity, which could be mediated by PTEN loss or mutations (*e.g.* PC3 cells [36]), lack of inhibitory dephosphorylation of PI3K (*e.g.* HEK293 [37]), expression of IGBP1 (*e.g.* αT3-1 [21] or other regulatory mechanisms that still need clarification. We then studied the mechanism that induces the immediate AKT dephosphorylation, and found that it is mediated primarily by PP2A without effects of PTEN or other phosphatases [22]. We further found that the mechanism by which PP2A reduces AKT activity in these cells involves the non-canonical regulatory subunit IGBP1 [22]. In resting cells, IGBP1 binds to PP2Ac, and this dimer interacts with the regulatory subunit of PI3K and dephosphorylates the auto-inhibitory pSer608-p85 subunit. This dephosphorylation maintains high basal activity of the PI3K, and the phosphoinositide molecules produced recruit AKT to the plasma membrane where it is phosphorylated and activated. Upon GqPCR activation or by a direct activation of PKC, the PP2Ac-IGBP1 dimer is detached from p85-PI3K. The lack of attached phosphatase allows accumulation of the phosphate at that location and therefore inhibition of PI3K [22], leading to reduced levels of phosphoinositide. This is followed by detachment of pAKT from the plasma membrane and its accumulation in the cytoplasm, a process that may lead to dephosphorylation of the kinase [38]. In parallel, the IGBP direct both PP2Ac and PP2Aa to the cytoplasmic pAKT and causes dephosphorylation and inactivation of the latter. As mentioned above, other B subunits can direct PP2Ac to AKT in the cytoplasm, but IGBP1 is the major component playing a role in our system [22]. This mechanism mediates an inhibitory action on both components of the cascade, PI3K and AKT, and this transfer from PI3K to AKT was termed: “PP2A switch”.

**Fig. 8:**
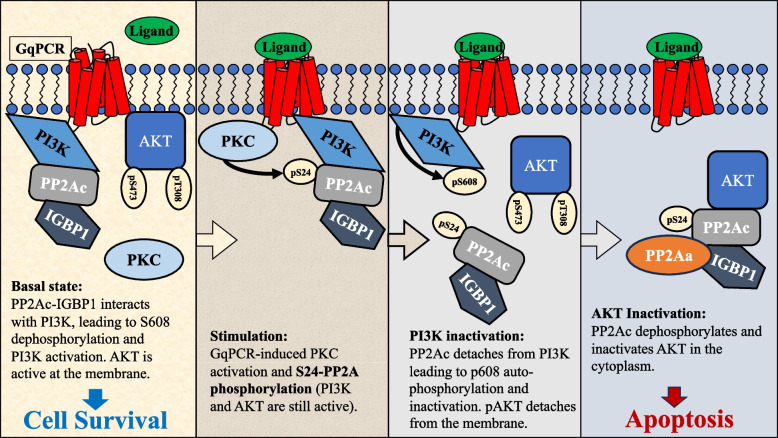
Schematic representation of the PKC-induced PP2Ac phosphorylation in the induction of the PP2A switch. In resting cells (first panel), the PP2Ac-IGBP1 dimer binds PI3K, dephosphorylating its inhibitory phosphorylation site (Ser608-p85-PI3K), leading to its activation and consequently cell survival. Upon GqPCR stimulation, PKC phosphorylates PP2Ac on Ser24 residue (second panel). This leads to the release of the IGBP1-PP2Ac dimer form PI3K (third panel), leading to autophosphorylation of PI3K, its inactivation and the detachment of AKT from the plasma membrane. The free PP2Ac-IGBP1 dimer together with PP2Aa bind to the cytoplasmic AKT leading to AKT dephosphorylation, inactivation and consequently cell death (Fourth panel)

The question that was still unsolved by the two previous reports was the mechanism that induces the PP2A switch upon stimulation. Here we found that this is mediated by phosphorylation of Ser 24 of PP2Ac by PKCα and to some extent also PKCβ. Our results best fit a model (depicted in Fig. 8) in which PP2Ac-IGBP1 heterodimer constitutively interacts with PI3K and dephosphorylates its inhibitory site (pSer608) in resting cells. Upon stimulation, PKC is recruited to the plasma membrane where it comes to close proximity with the PI3K-bound PP2Ac and phosphorylates it on Ser24-PP2Ac residue. This phosphorylation then induces the detachment of the IGBP1-PP2Ac dimer from PI3K and thereby enables the autophosphorylation of the inhibitory Ser608 of p85-PI3K, which result in the PI3K inactivation. Thereafter, the PP2Ac-IGBP1 dimer, forms a heterotrimer with PP2Aa, which interacts with AKT, and reduces the phosphorylation and activity of this kinase as well. This inactivation consequently results in the activation of JNK1/2 which in turn, leads to apoptosis. Interestingly, we found here that expression of S24E-PP2Ac (mimicking constitutive phosphorylation) is able to induce a constant and robust apoptosis of the cell lines examined, indicating that ser24-PP2Ac phosphorylation by classical PKCs is probably necessary and sufficient to induce the PP2A switch upon GqPCR stimulation in various cell lines.

Here we identified the primary step in the induction of the PP2A switch as the phosphorylation of PP2Ac by PKC that eventually leads to apoptosis by inhibiting AKT phosphorylation. PP2A regulates numerous cellular signaling pathways that lead to development, cell cycle, proliferation, apoptosis and more. The involvement of PP2A in apoptosis is relatively well studied [9] although the mechanisms identified thus far are clearly distinct from the PP2A-switch that we identified. Our findings show that the PP2A catalytic subunit, PP2Ac is phosphorylated on Ser24 in order to induce the PP2A switch that leads eventually to AKT inactivation. This is the first identification of this particular modification, although several other post-translational modifications such as methylation, degradation and phosphorylation have already been shown to regulate PP2Ac activity, stability, and holoenzyme biogenesis such as methylation and phosphorylation [39].

Our results clearly present a unique regulation of PP2A by phosphorylation. However, PP2Ac was previously shown to be regulated by other post-translational modifications. One of the first modification identified was methylation at Leu 309 in the C terminus of PP2Ac which is mediated by LCMT1 [40] and PME1, that activates PP2Ac, and enhances trimeric holoenzyme assembly [41, 42]. This methylation is essential for the formation in some (e.g. B55, B56 and B72; [43]) B subunits. On the other hand, in the case of PP2A/PR55a, the methylation was shown to be important for PP2A holoenzyme disassembly that leads to tau hyperphosphorylation and cell death in neurodegenerative diseases [44, 45].

As for phosphorylation, it was initially thought that due to the very efficient phosphatase activity of PP2A, this enzyme could not be stably phosphorylated, as it removes any phosphate incorporated. However, over the years it was shown that the different subunits of the PP2A do undergo regulatory phosphorylations that affect their activity. The C-terminal tail of PP2Ac was shown to be a substrate for post-translational modifications that modulate its interaction with specific B subunits and thus affect holoenzyme assembly and PP2A specificity [46]. Although the identity of the protein kinases has remained unknown, phosphorylation of Thr304 [47–50] regulates PP2A inactivation, mainly by inhibiting holoenzyme assembly. It was shown that binding of PP2Ac to B55 is dependent on Tyr 304 phosphorylation by CDK1, resulting in decreased dephosphorylation of PP2A/B55 substrates that promote mitotic entry [46]. Phosphorylation of Tyr 307 of PP2Ac disrupted its binding to PP2A regulatory subunit PR61/B’ [51, 52]. This phosphorylation also indirectly affects the assembly of PP2A with PR55/ B by preventing Leu309 methylation due to impaired access to the LCMT1 cavity [47, 51–53]. Thus, all these modifications in the C terminus of PP2Ac impair PP2A activity via disruption of PP2Ac binding to a regulatory PP2A B subunit. Other tyrosine phosphorylation of PP2A have been reported as well (http://www.phosphonet.ca/default.aspx#InfoBox), but the role of these phosphorylation is not entirely understood yet. In addition, various B subunits are regulated by phosphorylation as well. For example, it has been demonstrated that most of the B56 family members are phosphoproteins [54]. Interestingly, one of the protein kinases that induces such phosphorylations is PKC, which phosphorylates B56α on Ser41 [55]. All these modifications are distinct from our findings that show that phosphorylation of Ser24 residue of PP2Ac indeed dissociated it from its substrate (PI3K) albeit does not affect its activity but rather initiate the PP2A switch by rendering it accessible for AKT binding.

Our results here indicate that the PP2A switch-induced apoptosis is initiated by PKC phosphorylation of PP2Ac. PKC is a group of nine gene products that encode similar protein kinases, which can be devided into conventional (α, β, γ) novel (δ, ε, η, θ) and atypical (ζ,ι) isoforms [56, 57]. Although PKCs are better known for their ability to induce survial/proliferation or, when dysregulated, promote cancer [58], they were shown to participate in some growth inhibition, apoptosis, and tumor suppressing processes [59, 60]. In the current study we show that PKC may induce apoptosis of some cells by inhibiting AKT phosphorylation via PP2Ac. To the best of our knowledge, similar activity of PKC has not been reported in other system. However, induction of apoptosis by PKC via other mechanism has been reported in various cell line by several mechanisms. Interestingly, the main PKC suggested to be involved the induction of apoptosis is PKCδ [61], but we have shown that this PKC is not involved in the PP2A switch (Fig. S2). Interestingly, PKCα and PKCβ that do play a role in the induction of the PP2Ac switch are ususally involved in cell survival, and proliferation [60, 62]. Nonetheless these isoforms are still able to induce apoptosis in some cell lines following stimulation [62, 63]. For example, PKCα and PKCβII were shown to contribute to ROS-induced apoptosis of VSMCs [64] and PKCα was shown to induce apoptosis by downregulating the prosurvival mitochondrial protein Bcl2 [65]. Importantly, it was shown that PKCα can act as a tumor suppressor in PI3K/AKT-induced endometrial cancer, operating via PP2A [66]. Although the exact mechanism was not studied in that report, it is possible that it is similar to the system identified here. It is likely that this number of systems in which PKCα/β are involved in apoptosis will increase because Gq was shown to play a role in apoptosis in some cell types 10.3390/ijms241713527, but these are still limited as in a screen that we made, such system was identified in less then 15% of the cells examined [21].

## Conclusions

Our data support a mechanism depicted in Fig. 8 in which binding of GqPCR to its ligand in several cell types activates PKCα/β, which in turn bind and phosphorylate PI3K-bound PP2Ac on its serine 24 residue. This event induces release of the PP2Ac-IGBP1 dimer from PI3K, resulting in PI3K autophosphorylation on its inhibitory serine 608 of the p85 subunit. The free dimer forms a trimer with PP2Aa, binds and inactivates AKT via dephosphorylation. Since serine 24 phosphorylation is necessary and sufficient for conferring cellular apoptosis even in non-stimulated prostate cancer cells, further study on its possible use as a potential antitumor drug is needed.

### Supplementary Information

**Additional file 1: Figure S1.** Characterization of the anti pS24-PP2Ac Ab. **Figure S2.**Activated PKCa and PKCb1 isoforms bind and phosphorylate PP2Ac. **Figure S3.** PP2Ac mutants do not affect the catalytic PP2Ac activity. **Figure S4.** Using PLA to follow PP2Ac interaction with AKT, PI3K and IGBP1. **Figure S5.** IGBP1 binds PP2Aa indirectly dependent on Ser24-PP2Ac phosphorylation. **Figure S6.** The PP2A switch-related phosphorylation inhibits activation of the AKT substrate FOXO1.

## Data Availability

All data and materials are described in the text.

## Abbreviations

GnRH: Gonadotropin-releasing hormone
GPCR: G protein coupled receptors
IGBP: Immunoglobulin binding protein
PGF2α: Prostaglandin F2α
PI3K: Phosphoinositol-3 kinase
PKC: Protein kinase C
PP2A: Protein phosphatase 2A
TUNEL: Deoxynucleotidyltransferase-mediated nick end labeling
