# Supplementary material for: Phosphorylation of PP2Ac by PKC is a key regulatory step in the PP2A-switch-dependent AKT dephosphorylation that leads to apoptosis
Source: Cell Commun Signal. 2024 Feb 28;22:154. doi: 10.1186/s12964-024-01536-7 (PMC10900696; doi:10.1186/s12964-024-01536-7)
Supplement: Supplementary file 1 — Additional file 1: Figure S1. Characterization of the anti pS24-PP2Ac Ab. Figure S2.Activated PKCa and PKCb1 isoforms bind and phosphorylate PP2Ac. Figure S3. PP2Ac mutants do not affect the catalytic PP2Ac activity. Figure S4. Using PLA to follow PP2Ac interaction with AKT, PI3K and IGBP1. Figure S5. IGBP1 binds PP2Aa indirectly dependent on Ser24-PP2Ac phosphorylation. Figure S6. The PP2A switch-related phosphorylation inhibits activation of the AKT substrate FOXO1. [file 12964_2024_1536_MOESM1_ESM.pdf]

Supplementary Figures

Phosphorylation of PP2Ac by PKC is a key regulatory step in the PP2A-switch-dependent AKT dephosphorylation that lead to apoptosis

Guy Nadel, Zhong Yao, Avital Hacohen-Lev-Ran, Ehud Wainstein, Galia Maik-Rachline,

Tamar Ziv, Zvi Naor, Arie Admon, and Rony Seger

Figures

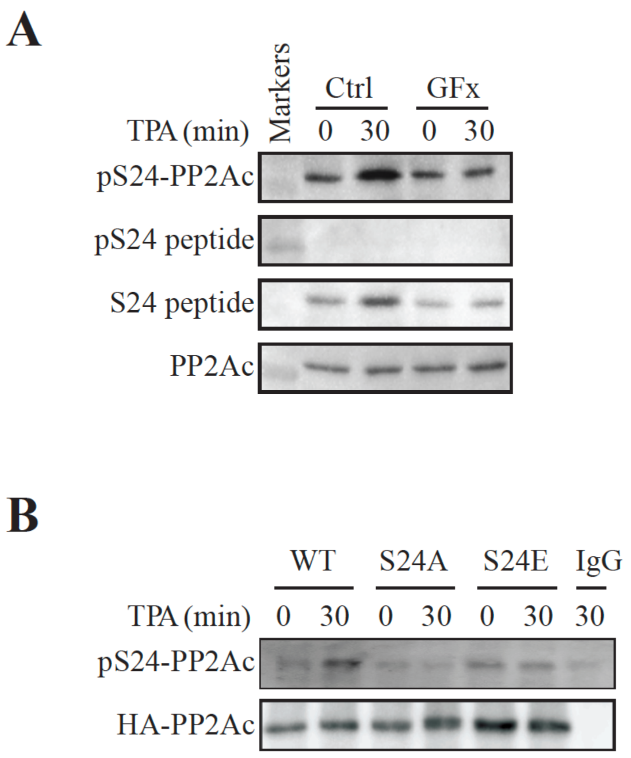

Figure S1. Characterization of the anti pS24-PP2Ac Ab. (A) Specificity of the Ab. PC3 cells were grown to 70% confluence, starved (0.1% FCS, 16h) and treated with GF109203x for 20 min prior to stimulation with TPA (250 nM, 30 min; (+) or DMSO control (-)). Then the cells were harvested separated by SDS-PAGE and immunoblotted with the anti pS24-PP2Ac Ab . In parallel, the immunogenic peptide used to generate the pSer24-PP2Ac Ab (pS24 peptide, second panel), and the same peptide without phosphate (S24 peptide, third panel; both at 100 µg/ml) were incubated with the anti pS24 -PP2Ac Ab to compete out specific interactions (1h, 23°C). Then each of the Ab treated with the distinct peptides was used to immunoblot the same extracts. The amount of PP2Ac is seen in the lower panel using anti PP2A Ab. (**B**). The pSer24-PP2Ac Ab is specific to the phosphorylated residue. αT3-1 cells were transfected with either HA-PP2Ac or its phospho site mutants (HA-S24A, and HA-S24E), in serum starvation medium (0.1% FCS, 16h). The cells were then either stimulated with TPA (250 nM 30 min; (+) or left untreated (-)). The cells were harvested, the PP2Ac mutants were IPed using anti-HA Ab, and detected with either pS24-PP2Ac (upper panel) or HA (lower panel) Abs.

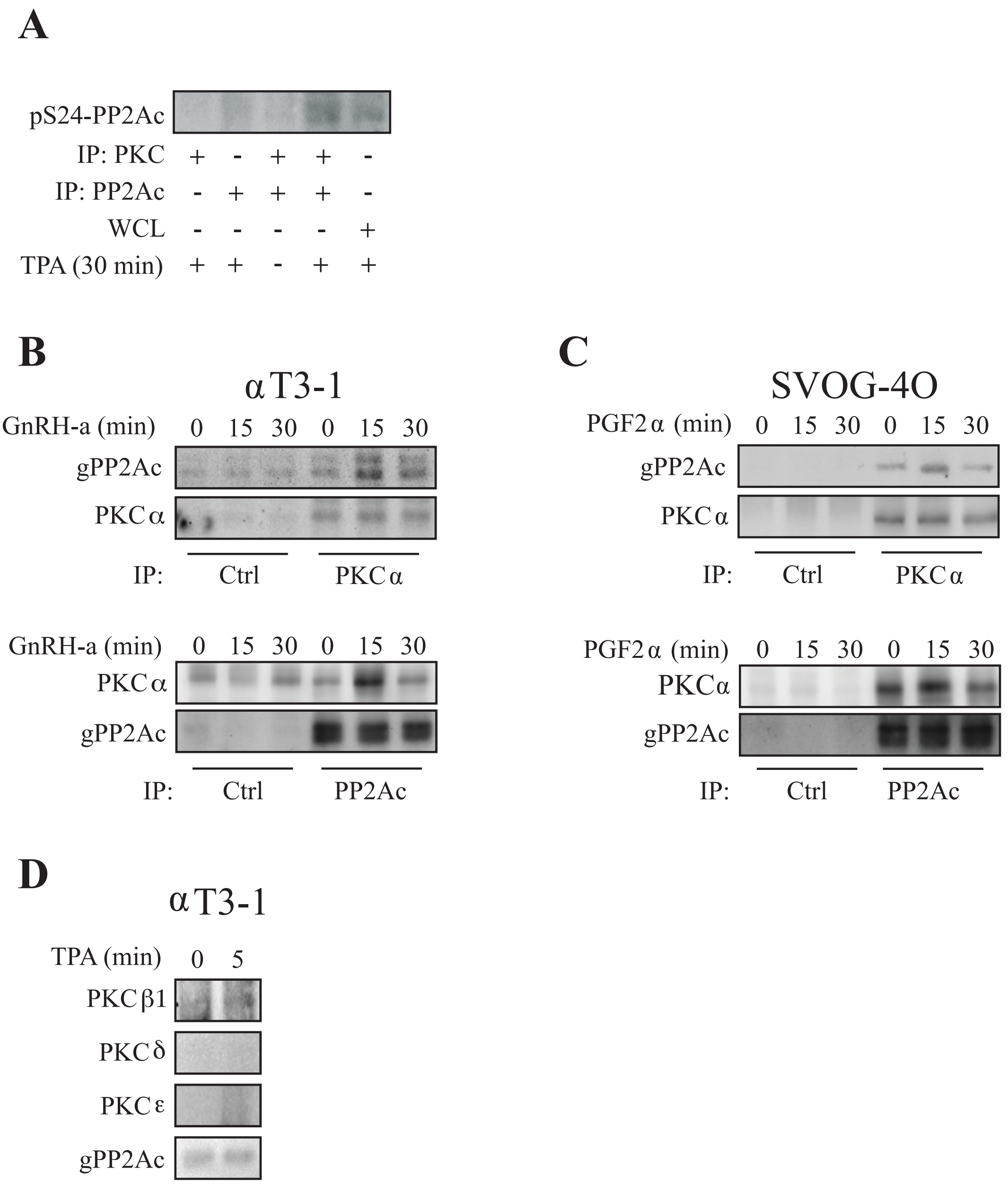

Figure S2. Activated PKCα and PKCβ1 isoforms bind and phosphorylate PP2Ac. (**A**). In vitro phosphorylation of PP2Ac-S24 by PKCα. Serum starved (0.1% FCS, 16h) PC3 cells were either stimulated with TPA (250 nM) for the indicated times (+) or left untreated (-). The cells were then harvested, followed by IP of PKCα. Additional serum starved PC3 were harvested, and PP2Ac was IPed. The precipitated proteins were co-incubated in 37^0C for 1h, resolved on SDS-PAGE and pS24-PP2Ac Ab was used to detect in vitro phosphorylation events. **(B,C)** PKCα binds PP2Ac after GqPCR stimulation. αT3-1 (B) or SVOG-4O (C) were serum starved (0.1% FCS, 16h) and the cells were stimulated with GnRH-a or PGF2α for the indicated times. The cells were then harvested, followed by IP of PKCα (upper panels) or PP2Ac (lower panels). The bound proteins were detected by Western blotting with the relevant Abs. (**D**). PKCβ1, but not PKCδ and PKCε binds PP2Ac after TPA stimulation. Serum starved (0.1% FCS, 16h) αT3-1 cells were stimulated with 250 nM TPA for the indicated times. The cells were then harvested, PP2Ac was IPed and the interaction of PKC isoforms was detected by immunoblotting.

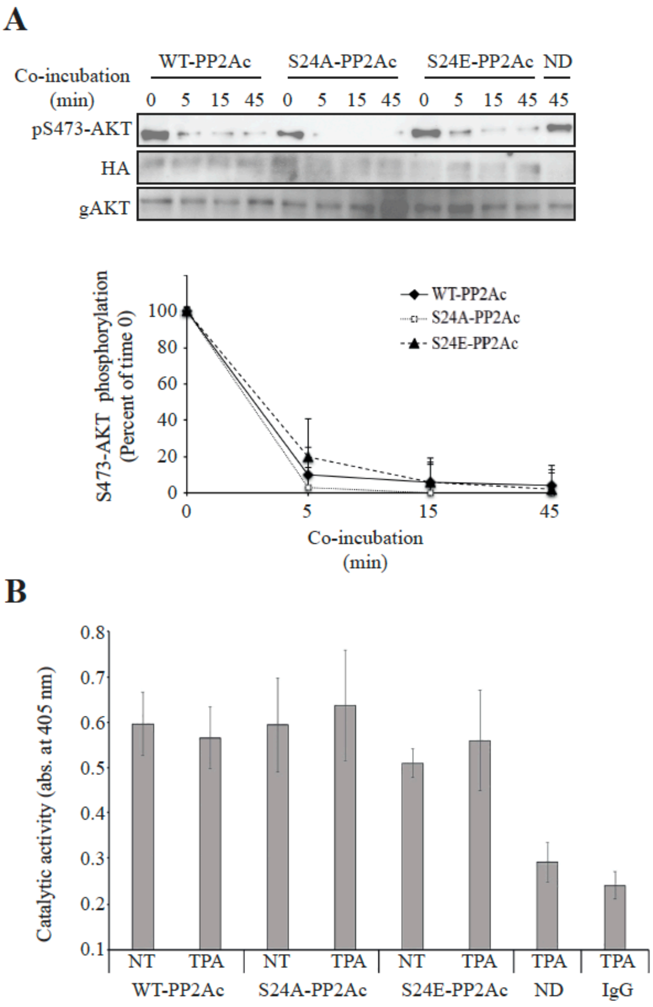

Figure S3. PP2Ac mutants do not affect the catalytic PP2Ac activity. (A). In vitro AKT dephosphorylation by PP2Ac mutants was measured essentially as described in Wasserman JS. et al. [64]. Briefly, PC3 cells were transfected with either the HA-PP2Ac constructs (HA-PP2Ac WT, S24A, S24E) or GFP-AKT1. Serum straved (0.1% FCS, 16h) cells transfected with HA-PP2Ac were treated with 250 nM TPA (250 nM, 30 minutes, while serum starved cells transfected with GFP-AKT1 were left untreated, and therefore phosphorylated on S473 as shown in [22]. Cells were harvested in "harvesting DIP buffer" 50 mM HEPES pH 7.2, 150 mM NaCl, 1 mM EDTA, 2.5 mM EGTA, 10% glycerol, 1 mM DTT, 1 ug/ml Aprotinin, 1 mM Benzamidine, 1 µg/ml Pepstatin A, 500 µM PMSF. After harvesting the cells were sonicated (50 w 2x7 seconds) and centrifuged (14,000xg 15 min). Next, each of the constructs was immunoprecipitated by the relevant Abs (anti HA for HA-PP2Ac and anti GFP for GFP-AKT1). The proteins-containing beads were washed three times in DIP buffer (50 mM HEPES pH 7.2, 150 mM NaCl, 1 mM EDTA, 2.5 mM EGTA, 10% glycerol, 1 mM DTT) and then the beads contain GFP-AKT1 were incubated with the beads of each of the constructs, for the times indicated in the same buffer. Dephosphorylation was then terminated by adding sample buffer and boiling. The samples were resolved by SDS Page and analyzed by the indicated Abs. The bar graphs in the lower panels show change in pS473-AKT1, calculated as percent of phosphorylation at time 0. The results represent means ± standard deviation (SD) of three experiments. (B) PC3 cells were grown, serum starved (0.1% FCS, 16h) and transfected either with HA-PP2Ac constructs or left with no DNA. The transfected cells were serum starved (0.1% FCS, 16h) and either left untreated (0) or stimulated with TPA (250 nM, 30 min). The

cells were then harvested with harvesting DIP buffer and the HA-PP2Ac was IPed. IgG was used as a Ab control of WT transfected TPA stimulated cells. Then the IPed proteins were washed (x3) by DIP buffer followed by one wash with HEPES buffer (50 mM pH 7.8 with 1 mM DTT). The beads were then suspended in a reaction buffer (50mM HEPES pH 7.8, 10 mM $MgCl_2$ and 1mM DTT) and incubated with the substrate pNPP, (53.8 mM, total volume 100 µm). The reaction was by appearance of yellow color detected (17h) at 405 nm in 300C. The bar-graph represents absorbance values above control (no cell lysate). Data are means ± SD. No significant changes were detected in both A and B.

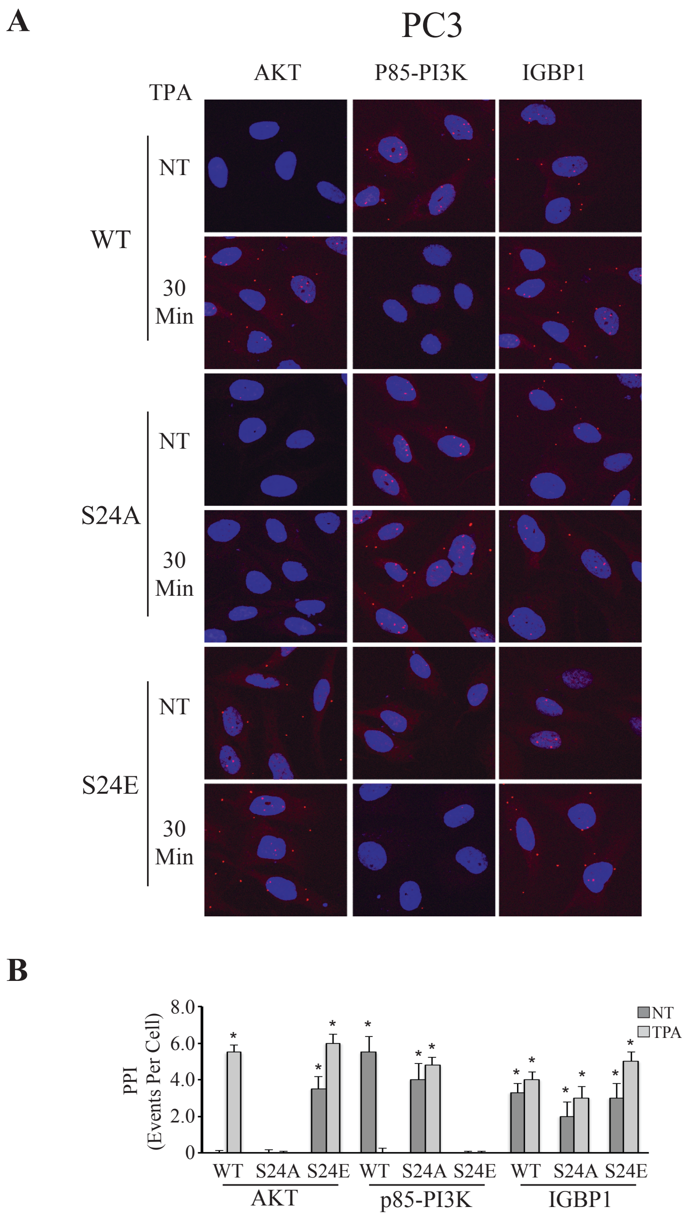

Figure S4. Using PLA to follow PP2Ac interaction with AKT, PI3K and IGBP1. PC3 cells were cultured on cover slips. The cells were transfected with either HA-WT-PP2Ac or PP2Ac mutants (S24A, S24E) in PCDNA3, in serum starvation medium (0.1% FCS, 16h). The cells were then either stimulated with TPA (250 nM, 30 min) or left untreated (NT) and then fixed. Protein-protein interactions were detected using the PLA kit with anti HA for PP2Ac isoforms together with each of the other specific Abs (AKT, p85-PI3K and IGBP1), as described under Methods. The bar graphs represent means ± SD of a representative experiment that was reproduced 3 times. Significance of change from non-stimulated (NT) is calculated. * $p<0.01$.

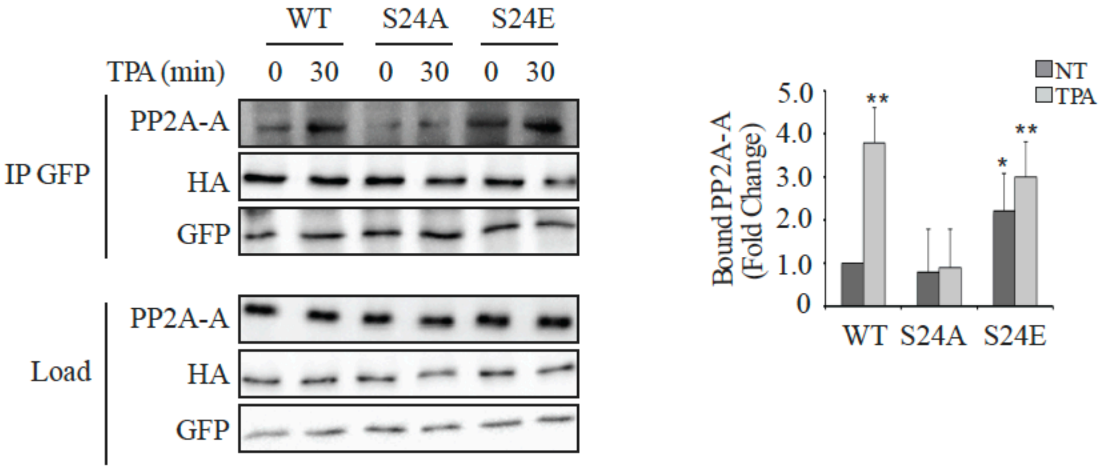

Figure S5. IGBP1 binds PP2Aa indirectly dependent on Ser24-PP2Ac phosphorylation. PC3 cells were transfected with GFP-IGBP1 and either HA-PP2Ac or PP2Ac mutants (S24A, S24E) in PCDNA3 in serum starvation medium (0.1% FCS, 16h) and then were either stimulated with TPA (250 nM, 30 min) or left untreated. Cells were then harvested and GFP-IGBP1 was IPed in a CoIP experiment using anti GFP Ab. The CoIPed proteins were analyzed using the indicated Abs. Stimulated cells transfected with GFP-IGBP1 were IPed with IgG control. The bar graph represents the amount of PP2Aa divided by the amount of HA. This is an average of three experiments ± SD. Significance of change from non-stimulated WT was calculated. * $p<0.01$

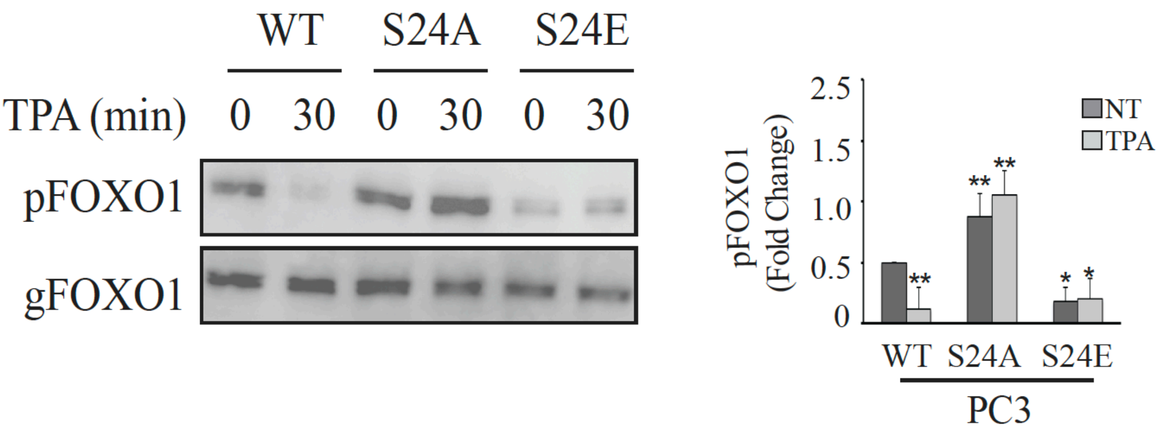

Figure S6. **The PP2A switch-related phosphorylation inhibits activation of the AKT substrate FOXO1.** PC3 cells were transfected with either HA-WT-PP2Ac or PP2Ac mutants (S24A, S24E) in PCDNA3 in serum starvation medium (0.1% FCS, 16h), and then either stimulated by TPA (250 nM, 30 min) or left untreated (0). Cells were harvested and then cell extracts were analysed by Western blotting with the indicated Abs. The bar graphs represent means ± SD of a representative experiment that was reproduced 3 times. Significance of change from WT non-stimulated is calculated. * $p<0.01$.
